# 2,2-Diphenyl-4-(piperidin-1-yl)butanamide

**DOI:** 10.1107/S1600536811024585

**Published:** 2011-06-25

**Authors:** M. S. Siddegowda, Jerry P. Jasinski, James A. Golen, H. S. Yathirajan, M. T. Swamy

**Affiliations:** a Department of Studies in Chemistry, University of Mysore, Manasagangotri, Mysore 570 006, India; b Department of Chemistry, Keene State College, 229 Main Street, Keene, NH 03435-2001, USA; c Department of Chemistry, Sambhram Institute of Technology, Bangalore 560 097, India

## Abstract

In the title compound, C_21_H_26_N_2_O, the dihedral angle between the mean planes of the two benzene rings is 81.1 (9)°. The piperidine ring is in a chair conformation. The crystal packing is stabilized by N—H⋯N and N—H⋯O hydrogen bonds and weak inter­molecular C—H⋯O inter­actions.

## Related literature

For the biological activity and pharmaceutical applications of compounds similar to the title compound, see: Guzel *et al.* (2006 ▶). For related structures, see: Akkurt *et al.* (2007 ▶); Dutkiewicz *et al.* (2010 ▶); Gerkin (1998 ▶); Krigbaum *et al.* (1968 ▶); Narasegowda *et al.* (2005 ▶); Yathirajan *et al.* (2005 ▶). For standard bond lengths, see Allen *et al.* (1987 ▶). For puckering parameters, see: Cremer & Pople (1975 ▶).
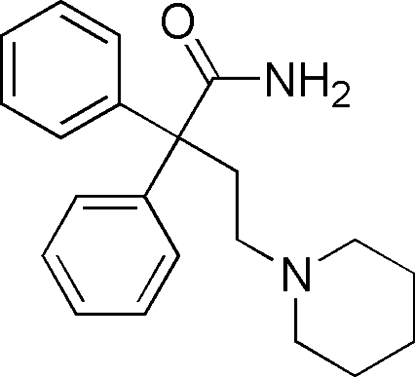


## Experimental

### 

#### Crystal data

C_21_H_26_N_2_O
*M* *_r_* = 322.44
Orthorhombic, 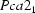

*a* = 18.1070 (12) Å
*b* = 10.3025 (9) Å
*c* = 9.6150 (6) Å
*V* = 1793.7 (2) Å^3^
*Z* = 4
Mo *K*α radiation
μ = 0.07 mm^−1^
*T* = 173 K
0.40 × 0.32 × 0.20 mm

#### Data collection

Oxford Diffraction Xcalibur Eos Gemini diffractometer
Absorption correction: multi-scan (*CrysAlis RED*; Oxford Diffraction, 2010 ▶) *T*_min_ = 0.971, *T*_max_ = 0.986
19279 measured reflections
4817 independent reflections
4547 reflections with *I* > 2σ(*I*)
*R*_int_ = 0.020

#### Refinement

*R*[*F* ^2^ > 2σ(*F* ^2^)] = 0.036
*wR*(*F* ^2^) = 0.102
*S* = 1.02
4817 reflections
224 parameters
4 restraints
H atoms treated by a mixture of independent and constrained refinement
Δρ_max_ = 0.22 e Å^−3^
Δρ_min_ = −0.16 e Å^−3^

### 

Data collection: *CrysAlis PRO* (Oxford Diffraction, 2010 ▶); cell refinement: *CrysAlis PRO*; data reduction: *CrysAlis RED* (Oxford Diffraction, 2010 ▶); program(s) used to solve structure: *SHELXS97* (Sheldrick, 2008 ▶); program(s) used to refine structure: *SHELXL97* (Sheldrick, 2008 ▶); molecular graphics: *SHELXTL* (Sheldrick, 2008 ▶); software used to prepare material for publication: *SHELXTL*.

## Supplementary Material

Crystal structure: contains datablock(s) global, I. DOI: 10.1107/S1600536811024585/sj5167sup1.cif

Structure factors: contains datablock(s) I. DOI: 10.1107/S1600536811024585/sj5167Isup2.hkl

Supplementary material file. DOI: 10.1107/S1600536811024585/sj5167Isup3.cml

Additional supplementary materials:  crystallographic information; 3D view; checkCIF report

## Figures and Tables

**Table 1:** Hydrogen-bond geometry (Å, °)

*D*—H⋯*A*	*D*—H	H⋯*A*	*D*⋯*A*	*D*—H⋯*A*
N1—H1*NB*⋯N2^i^	0.87 (1)	2.08 (1)	2.9457 (13)	177 (2)
N1—H1*NA*⋯O1^ii^	0.84 (1)	2.38 (1)	3.1971 (14)	164 (2)
C3—H3*B*⋯O1^ii^	0.99	2.47	3.3738 (14)	152

Symmetry codes: (i) 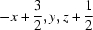; (ii) 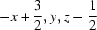.

**Fig. 1.**
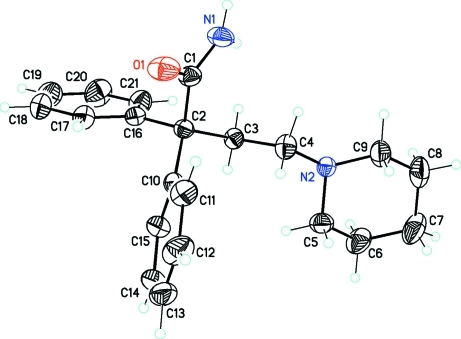
Molecular structure of the title compound showing the atom labeling scheme and 50% probability displacement ellipsoids.

**Fig. 2.**
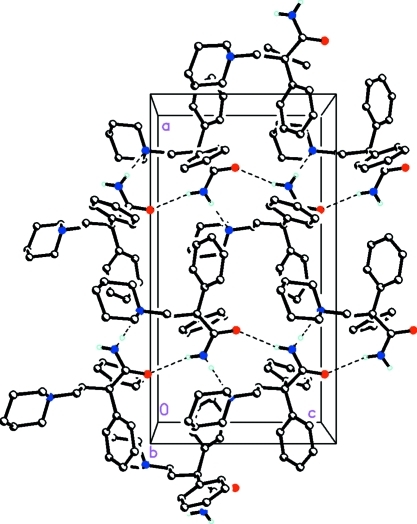
Packing diagram od the title compound viewed down the b axis. Dashed lines represent N—H···N, N—H···O hydrogen bonds and weak C—H···O intermolecular interactions.

## supplementary crystallographic information

### Comment

The title compound is an intermediate used in the synthesis of biologically and pharmaceutically active compounds viz., loperamide, darifenacin, fenpiverine, etc. The synthesis and antimycobacterial activity of some new related 2,2-diphenylacetamide derivatives has been described (Guzel *et al.*, 2006). The crystal structures of N,N-diphenylacetamide (Krigbaum *et al.*, 1968), 4,4'-dimethylbiphenyl-2,2'-dicarboxylic acid (Gerkin, 1998), 4'-methylbiphenyl-2-carboxylic acid (Narasegowda *et al.*, 2005) and 4'-(2-butyl-4-chloro-5-formylimidazol-1-ylmethyl) biphenyl-2-carbonitrile (Yathirajan *et al.*, 2005), 2-hydroxy-N-(3-oxo-1-thia-4-azaspiro[4.5]dec-4-yl)-2,2-diphenylacetamide (Akkurt *et al.*, 2007) and 2-chloro-N-[4-chloro-2-(2-chlorobenzoyl) phenyl]acetamide (Dutkiewicz *et al.*, 2010) have been reported. In view of the importance of the title compound, (I), C_21_H_26_N_2_O, a new crystal structure determination is reported.

In the title compound, (I), the dihedral angle between the mean planes of the two benzene rings is 81.1 (9)° (Fig. 1). The piperidin-1-yl ring is in a chair conformation (Cremer & Pople (1975), puckering parameters Q, θ, and φ = 0.5689 (15)A%, 3.89 (16)° and 14 (2)° respectively). For an ideal chair θ has a value of 0 or 180°. Bond lengths are normal (Allen *et al.*, 1987).  Crystal packing is stabilized by N—H···N, N—H···O hydrogen bonds and weak C—H···O intermolecular interactions (Fig. 2, Table 1).

### Experimental

The title compound was obtained as a gift sample from R. L. Fine Chem, Bangalore. X-ray quality crystals were obtained by slow evaporation of (1:1) methanol and dichloromethane  solution (m.p.: 458-460 K).

### Refinement

The N–H atoms were located by Fourier analysis and refined isotropically with DFIX = 0.86Å and DANG - 1.40Å.  All of the remaining H atoms were placed in their calculated positions and then refined using the riding model with Atom–H lengths of 0.95Å (CH), or 0.99Å (CH_2_).  Isotropic displacement parameters for these atoms were set to 1.18-1.20 (CH) or (CH_2_) times *U*_eq_ of the parent atom.

### Crystal data

**Table:**

C_21_H_26_N_2_O	*F*(000) = 696
*M**_r_* = 322.44	*D*_x_ = 1.194 Mg m^−^^3^
Orthorhombic, *P**c**a*2_1_	Mo *K*α radiation, λ = 0.71073 Å
Hall symbol: P 2c -2ac	Cell parameters from 9220 reflections
*a* = 18.1070 (12) Å	θ = 3.4–32.5°
*b* = 10.3025 (9) Å	µ = 0.07 mm^−^^1^
*c* = 9.6150 (6) Å	*T* = 173 K
*V* = 1793.7 (2) Å^3^	Block, colorless
*Z* = 4	0.40 × 0.32 × 0.20 mm

### Data collection

**Table:**

Oxford Diffraction Xcalibur Eos Gemini diffractometer	4817 independent reflections
Radiation source: Enhance (Mo) X-ray Source	4547 reflections with *I* > 2σ(*I*)
graphite	*R*_int_ = 0.020
Detector resolution: 16.1500 pixels mm^-1^	θ_max_ = 29.1°, θ_min_ = 3.7°
ω scans	*h* = −24→24
Absorption correction: multi-scan (*CrysAlis RED*; Oxford Diffraction, 2010)	*k* = −13→14
*T*_min_ = 0.971, *T*_max_ = 0.986	*l* = −13→13
19279 measured reflections	

### Refinement

**Table:**

Refinement on *F*^2^	Primary atom site location: structure-invariant direct methods
Least-squares matrix: full	Secondary atom site location: difference Fourier map
*R*[*F*^2^ > 2σ(*F*^2^)] = 0.036	Hydrogen site location: inferred from neighbouring sites
*wR*(*F*^2^) = 0.102	H atoms treated by a mixture of independent and constrained refinement
*S* = 1.02	*w* = 1/[σ^2^(*F*_o_^2^) + (0.0674*P*)^2^ + 0.1453*P*] where *P* = (*F*_o_^2^ + 2*F*_c_^2^)/3
4817 reflections	(Δ/σ)_max_ < 0.001
224 parameters	Δρ_max_ = 0.22 e Å^−^^3^
4 restraints	Δρ_min_ = −0.16 e Å^−^^3^

### Special details

**Table:**

Geometry. All esds (except the esd in the dihedral angle between two l.s. planes) are estimated using the full covariance matrix. The cell esds are taken into account individually in the estimation of esds in distances, angles and torsion angles; correlations between esds in cell parameters are only used when they are defined by crystal symmetry. An approximate (isotropic) treatment of cell esds is used for estimating esds involving l.s. planes.
Refinement. Refinement of *F*^2^ against ALL reflections. The weighted *R*-factor *wR* and goodness of fit *S* are based on *F*^2^, conventional *R*-factors *R* are based on *F*, with *F* set to zero for negative *F*^2^. The threshold expression of *F*^2^ > σ(*F*^2^) is used only for calculating *R*-factors(gt) *etc*. and is not relevant to the choice of reflections for refinement. *R*-factors based on *F*^2^ are statistically about twice as large as those based on *F*, and *R*- factors based on ALL data will be even larger.

### Fractional atomic coordinates and isotropic or equivalent isotropic displacement parameters (Å^2^)

**Table:**

	*x*	*y*	*z*	*U*_iso_*/*U*_eq_	
O1	0.68400 (5)	0.34583 (11)	0.96707 (9)	0.0432 (2)	
N1	0.75885 (6)	0.33139 (12)	0.78130 (11)	0.0375 (2)	
H1NB	0.7944 (8)	0.2941 (16)	0.8267 (16)	0.045*	
H1NA	0.7651 (9)	0.3416 (16)	0.6956 (14)	0.045*	
N2	0.62349 (5)	0.19366 (9)	0.43291 (9)	0.02659 (18)	
C1	0.69459 (6)	0.36080 (11)	0.84208 (11)	0.0287 (2)	
C2	0.63630 (5)	0.42567 (10)	0.74607 (9)	0.02408 (18)	
C3	0.64319 (6)	0.37719 (10)	0.59391 (10)	0.02621 (19)	
H3A	0.6058	0.4219	0.5361	0.031*	
H3B	0.6925	0.4011	0.5577	0.031*	
C4	0.63287 (7)	0.23102 (11)	0.57931 (11)	0.0318 (2)	
H4A	0.6764	0.1858	0.6183	0.038*	
H4B	0.5889	0.2036	0.6330	0.038*	
C5	0.54694 (6)	0.21203 (12)	0.38868 (13)	0.0356 (2)	
H5A	0.5320	0.3030	0.4065	0.043*	
H5B	0.5144	0.1547	0.4440	0.043*	
C6	0.53738 (9)	0.18176 (15)	0.23557 (15)	0.0470 (3)	
H6A	0.5671	0.2433	0.1797	0.056*	
H6B	0.4849	0.1928	0.2094	0.056*	
C7	0.56157 (11)	0.04385 (16)	0.20378 (18)	0.0579 (4)	
H7A	0.5270	−0.0182	0.2477	0.070*	
H7B	0.5606	0.0292	0.1020	0.070*	
C8	0.63853 (10)	0.02107 (16)	0.25826 (18)	0.0578 (4)	
H8A	0.6740	0.0726	0.2028	0.069*	
H8B	0.6513	−0.0718	0.2471	0.069*	
C9	0.64546 (8)	0.05852 (13)	0.41083 (16)	0.0438 (3)	
H9A	0.6138	0.0009	0.4677	0.053*	
H9B	0.6972	0.0465	0.4414	0.053*	
C10	0.55575 (5)	0.40102 (11)	0.78870 (11)	0.0274 (2)	
C11	0.53249 (7)	0.30304 (12)	0.87673 (13)	0.0371 (2)	
H11A	0.5678	0.2506	0.9235	0.044*	
C12	0.45683 (8)	0.28120 (16)	0.89694 (16)	0.0494 (3)	
H12A	0.4414	0.2148	0.9589	0.059*	
C13	0.40475 (7)	0.35405 (17)	0.82879 (16)	0.0498 (4)	
H13A	0.3537	0.3367	0.8417	0.060*	
C14	0.42691 (7)	0.45219 (15)	0.74177 (16)	0.0455 (3)	
H14A	0.3912	0.5038	0.6951	0.055*	
C15	0.50184 (6)	0.47598 (12)	0.72201 (13)	0.0361 (2)	
H15A	0.5167	0.5445	0.6621	0.043*	
C16	0.65362 (5)	0.57150 (11)	0.76043 (11)	0.0287 (2)	
C17	0.63429 (7)	0.63353 (13)	0.88404 (14)	0.0404 (3)	
H17A	0.6108	0.5856	0.9559	0.048*	
C18	0.64896 (8)	0.76405 (15)	0.90339 (19)	0.0528 (4)	
H18A	0.6355	0.8050	0.9882	0.063*	
C19	0.68294 (9)	0.83480 (15)	0.8005 (2)	0.0565 (4)	
H19A	0.6922	0.9248	0.8133	0.068*	
C20	0.70346 (9)	0.77430 (15)	0.6784 (2)	0.0544 (4)	
H20A	0.7272	0.8228	0.6073	0.065*	
C21	0.68962 (7)	0.64287 (13)	0.65859 (15)	0.0400 (3)	
H21A	0.7049	0.6018	0.5749	0.048*	

### Atomic displacement parameters (Å^2^)

**Table:**

	*U*^11^	*U*^22^	*U*^33^	*U*^12^	*U*^13^	*U*^23^
O1	0.0344 (4)	0.0706 (6)	0.0246 (4)	0.0036 (4)	−0.0032 (3)	0.0074 (4)
N1	0.0269 (4)	0.0567 (6)	0.0288 (5)	0.0099 (4)	−0.0038 (4)	−0.0007 (4)
N2	0.0269 (4)	0.0282 (4)	0.0246 (4)	0.0000 (3)	−0.0001 (3)	−0.0028 (3)
C1	0.0264 (5)	0.0341 (5)	0.0256 (5)	0.0012 (4)	−0.0037 (4)	0.0008 (4)
C2	0.0227 (4)	0.0304 (5)	0.0191 (4)	0.0022 (3)	−0.0005 (3)	−0.0003 (3)
C3	0.0299 (5)	0.0296 (5)	0.0192 (4)	0.0006 (4)	0.0005 (3)	−0.0002 (4)
C4	0.0431 (6)	0.0296 (5)	0.0228 (5)	0.0013 (4)	−0.0025 (4)	0.0018 (4)
C5	0.0285 (5)	0.0430 (6)	0.0352 (6)	0.0008 (4)	−0.0033 (4)	−0.0015 (5)
C6	0.0552 (8)	0.0479 (7)	0.0380 (6)	−0.0021 (6)	−0.0164 (6)	−0.0029 (6)
C7	0.0775 (11)	0.0478 (8)	0.0485 (8)	−0.0076 (7)	−0.0186 (7)	−0.0154 (6)
C8	0.0703 (10)	0.0462 (8)	0.0569 (9)	0.0083 (7)	−0.0041 (8)	−0.0266 (7)
C9	0.0501 (7)	0.0324 (6)	0.0490 (7)	0.0079 (5)	−0.0094 (6)	−0.0099 (5)
C10	0.0248 (4)	0.0335 (5)	0.0239 (4)	−0.0001 (4)	0.0009 (4)	−0.0062 (4)
C11	0.0374 (6)	0.0427 (6)	0.0311 (6)	−0.0058 (5)	0.0018 (4)	0.0010 (5)
C12	0.0445 (7)	0.0618 (8)	0.0419 (7)	−0.0220 (6)	0.0100 (5)	−0.0038 (6)
C13	0.0299 (6)	0.0715 (10)	0.0480 (7)	−0.0109 (6)	0.0058 (5)	−0.0213 (7)
C14	0.0272 (5)	0.0587 (8)	0.0506 (7)	0.0074 (5)	−0.0032 (5)	−0.0175 (6)
C15	0.0285 (5)	0.0397 (6)	0.0402 (6)	0.0047 (4)	−0.0014 (4)	−0.0032 (5)
C16	0.0245 (4)	0.0322 (5)	0.0293 (5)	0.0009 (4)	−0.0038 (4)	−0.0028 (4)
C17	0.0371 (6)	0.0442 (7)	0.0399 (7)	−0.0008 (5)	0.0004 (5)	−0.0117 (5)
C18	0.0443 (7)	0.0509 (8)	0.0632 (9)	0.0003 (6)	−0.0042 (6)	−0.0278 (7)
C19	0.0467 (8)	0.0382 (7)	0.0846 (12)	−0.0066 (6)	−0.0075 (8)	−0.0158 (7)
C20	0.0576 (9)	0.0411 (7)	0.0646 (10)	−0.0136 (6)	−0.0005 (7)	0.0018 (6)
C21	0.0440 (6)	0.0380 (6)	0.0379 (6)	−0.0065 (5)	0.0011 (5)	−0.0018 (5)

### Geometric parameters (Å, °)

**Table:**

O1—C1	1.2267 (14)	C8—H8A	0.9900
N1—C1	1.3368 (14)	C8—H8B	0.9900
N1—H1NB	0.867 (12)	C9—H9A	0.9900
N1—H1NA	0.838 (13)	C9—H9B	0.9900
N2—C5	1.4621 (14)	C10—C11	1.3830 (16)
N2—C9	1.4635 (14)	C10—C15	1.4002 (16)
N2—C4	1.4692 (13)	C11—C12	1.4017 (18)
C1—C2	1.5534 (13)	C11—H11A	0.9500
C2—C10	1.5362 (13)	C12—C13	1.372 (2)
C2—C16	1.5411 (14)	C12—H12A	0.9500
C2—C3	1.5509 (13)	C13—C14	1.372 (2)
C3—C4	1.5239 (15)	C13—H13A	0.9500
C3—H3A	0.9900	C14—C15	1.3918 (17)
C3—H3B	0.9900	C14—H14A	0.9500
C4—H4A	0.9900	C15—H15A	0.9500
C4—H4B	0.9900	C16—C21	1.3871 (17)
C5—C6	1.5147 (18)	C16—C17	1.3941 (16)
C5—H5A	0.9900	C17—C18	1.3832 (19)
C5—H5B	0.9900	C17—H17A	0.9500
C6—C7	1.518 (2)	C18—C19	1.375 (3)
C6—H6A	0.9900	C18—H18A	0.9500
C6—H6B	0.9900	C19—C20	1.380 (3)
C7—C8	1.507 (2)	C19—H19A	0.9500
C7—H7A	0.9900	C20—C21	1.390 (2)
C7—H7B	0.9900	C20—H20A	0.9500
C8—C9	1.522 (2)	C21—H21A	0.9500
			
C1—N1—H1NB	121.7 (11)	C9—C8—H8A	109.3
C1—N1—H1NA	121.3 (11)	C7—C8—H8B	109.3
H1NB—N1—H1NA	116.7 (15)	C9—C8—H8B	109.3
C5—N2—C9	109.79 (9)	H8A—C8—H8B	107.9
C5—N2—C4	110.75 (9)	N2—C9—C8	111.01 (12)
C9—N2—C4	110.90 (10)	N2—C9—H9A	109.4
O1—C1—N1	122.40 (10)	C8—C9—H9A	109.4
O1—C1—C2	122.01 (10)	N2—C9—H9B	109.4
N1—C1—C2	115.41 (9)	C8—C9—H9B	109.4
C10—C2—C16	109.30 (8)	H9A—C9—H9B	108.0
C10—C2—C3	105.96 (8)	C11—C10—C15	118.07 (10)
C16—C2—C3	112.46 (8)	C11—C10—C2	124.97 (10)
C10—C2—C1	114.55 (8)	C15—C10—C2	116.65 (10)
C16—C2—C1	103.17 (8)	C10—C11—C12	119.97 (12)
C3—C2—C1	111.55 (8)	C10—C11—H11A	120.0
C4—C3—C2	113.28 (8)	C12—C11—H11A	120.0
C4—C3—H3A	108.9	C13—C12—C11	121.18 (14)
C2—C3—H3A	108.9	C13—C12—H12A	119.4
C4—C3—H3B	108.9	C11—C12—H12A	119.4
C2—C3—H3B	108.9	C12—C13—C14	119.56 (12)
H3A—C3—H3B	107.7	C12—C13—H13A	120.2
N2—C4—C3	111.18 (8)	C14—C13—H13A	120.2
N2—C4—H4A	109.4	C13—C14—C15	119.87 (13)
C3—C4—H4A	109.4	C13—C14—H14A	120.1
N2—C4—H4B	109.4	C15—C14—H14A	120.1
C3—C4—H4B	109.4	C14—C15—C10	121.33 (12)
H4A—C4—H4B	108.0	C14—C15—H15A	119.3
N2—C5—C6	111.37 (11)	C10—C15—H15A	119.3
N2—C5—H5A	109.4	C21—C16—C17	118.48 (11)
C6—C5—H5A	109.4	C21—C16—C2	123.31 (10)
N2—C5—H5B	109.4	C17—C16—C2	118.18 (10)
C6—C5—H5B	109.4	C18—C17—C16	120.80 (14)
H5A—C5—H5B	108.0	C18—C17—H17A	119.6
C5—C6—C7	110.82 (12)	C16—C17—H17A	119.6
C5—C6—H6A	109.5	C19—C18—C17	120.31 (15)
C7—C6—H6A	109.5	C19—C18—H18A	119.8
C5—C6—H6B	109.5	C17—C18—H18A	119.8
C7—C6—H6B	109.5	C18—C19—C20	119.56 (14)
H6A—C6—H6B	108.1	C18—C19—H19A	120.2
C8—C7—C6	110.04 (12)	C20—C19—H19A	120.2
C8—C7—H7A	109.7	C19—C20—C21	120.52 (16)
C6—C7—H7A	109.7	C19—C20—H20A	119.7
C8—C7—H7B	109.7	C21—C20—H20A	119.7
C6—C7—H7B	109.7	C16—C21—C20	120.29 (13)
H7A—C7—H7B	108.2	C16—C21—H21A	119.9
C7—C8—C9	111.82 (13)	C20—C21—H21A	119.9
C7—C8—H8A	109.3		
			
O1—C1—C2—C10	−32.45 (15)	C3—C2—C10—C15	−68.02 (12)
N1—C1—C2—C10	152.28 (10)	C1—C2—C10—C15	168.56 (10)
O1—C1—C2—C16	86.24 (13)	C15—C10—C11—C12	0.03 (17)
N1—C1—C2—C16	−89.03 (11)	C2—C10—C11—C12	−173.31 (11)
O1—C1—C2—C3	−152.81 (11)	C10—C11—C12—C13	1.2 (2)
N1—C1—C2—C3	31.92 (13)	C11—C12—C13—C14	−1.6 (2)
C10—C2—C3—C4	−65.67 (11)	C12—C13—C14—C15	0.8 (2)
C16—C2—C3—C4	174.97 (8)	C13—C14—C15—C10	0.40 (19)
C1—C2—C3—C4	59.61 (12)	C11—C10—C15—C14	−0.80 (17)
C5—N2—C4—C3	−82.56 (11)	C2—C10—C15—C14	173.09 (11)
C9—N2—C4—C3	155.28 (10)	C10—C2—C16—C21	−133.26 (11)
C2—C3—C4—N2	167.45 (9)	C3—C2—C16—C21	−15.87 (14)
C9—N2—C5—C6	−60.68 (14)	C1—C2—C16—C21	104.46 (12)
C4—N2—C5—C6	176.51 (10)	C10—C2—C16—C17	48.96 (12)
N2—C5—C6—C7	57.66 (16)	C3—C2—C16—C17	166.35 (10)
C5—C6—C7—C8	−52.61 (19)	C1—C2—C16—C17	−73.32 (11)
C6—C7—C8—C9	52.17 (19)	C21—C16—C17—C18	1.61 (19)
C5—N2—C9—C8	59.41 (15)	C2—C16—C17—C18	179.50 (11)
C4—N2—C9—C8	−177.87 (12)	C16—C17—C18—C19	0.0 (2)
C7—C8—C9—N2	−56.18 (18)	C17—C18—C19—C20	−1.1 (2)
C16—C2—C10—C11	−133.19 (11)	C18—C19—C20—C21	0.4 (3)
C3—C2—C10—C11	105.40 (11)	C17—C16—C21—C20	−2.26 (19)
C1—C2—C10—C11	−18.01 (15)	C2—C16—C21—C20	179.98 (12)
C16—C2—C10—C15	53.39 (12)	C19—C20—C21—C16	1.3 (2)

### Hydrogen-bond geometry (Å, °)

**Table:**

*D*—H···*A*	*D*—H	H···*A*	*D*···*A*	*D*—H···*A*
N1—H1NB···N2^i^	0.87 (1)	2.08 (1)	2.9457 (13)	177.(2)
N1—H1NA···O1^ii^	0.84 (1)	2.38 (1)	3.1971 (14)	164.(2)
C3—H3B···O1^ii^	0.99	2.47	3.3738 (14)	152

Symmetry codes: (i) −*x*+3/2, *y*, *z*+1/2; (ii) −*x*+3/2, *y*, *z*−1/2.
